# Prescribing Complexities: A Patient Story Related to Seizure History and the Changing Therapeutic Landscape of Alzheimer’s Disease

**DOI:** 10.7759/cureus.65127

**Published:** 2024-07-22

**Authors:** Mfon E Umoh, Samuel W Terman

**Affiliations:** 1 Medicine, Johns Hopkins University School of Medicine, Baltimore, USA; 2 Neurology, University of Michigan, Ann Arbor, USA

**Keywords:** risk-benefit considerations, medications, antiseizure medication, seizures, alzheimer’s disease

## Abstract

The therapeutic landscape of Alzheimer’s disease (AD) is rapidly changing. Disease-modifying medications for AD that target amyloid-beta (Aß) deposits in the brain have been approved by the Food and Drug Administration (FDA) in recent years. However, there remain many questions about which patients are most appropriate for these medications. One group in particular with unique considerations includes older adults with a prior history of seizures. AD and seizures represent an important, bidirectional relationship. This case report presents a patient story that highlights the importance of discussions around seizure history in consideration of anti-amyloid medications and the importance of risk-benefit assessments when considering anti-amyloid therapeutics for patients with AD.

## Introduction

Alzheimer's disease (AD) is the most prevalent neurodegenerative disorder in older people globally, and its therapeutic landscape is rapidly changing. Disease-modifying therapies that target and clear amyloid-beta (Aß) deposits in the brain have changed the clinical options available for patients with this ultimately progressive and devastating condition [1,2]. The US Food and Drug Administration (FDA) approved aducanumab, an anti-amyloid antibody, for early-stage AD in 2021 and lecanemab for mild cognitive impairment (MCI) and mild dementia due to AD in 2023. Most recently, in July 2024, the FDA approved donanemab for AD patients with MCI, or mild dementia. While many groups were underrepresented or not included in clinical trials for these medications, one group in particular with unique considerations includes older adults with a prior history of seizures.

Several exclusion criteria limit the use of monoclonal anti-amyloid medications [3,4]. Patients with a seizure history are excluded given the potential for amyloid-related imaging abnormalities (ARIA), a side effect of this new medication class, that can present with serious and life-threatening brain swelling or microhemorrhages that can be associated with seizures [5]. This exclusion is meant to minimize patient risk. A patient experience described below highlights the complexities of excluding patients with seizures. While AD and seizures are common conditions in older adults, AD is also a potential risk factor for the development of seizures [6]. This case highlights the importance of considering a patient's seizure history in decisions surrounding anti-amyloid therapies in patients with AD.

## Case presentation

A patient, in her sixth decade of life, came to the clinic with her partner. They were searching for anything that might help her memory. Her symptoms started gradually with word-finding difficulties, and she had experienced memory loss for the past 1.5 years. She was terrified because her mother and grandmother suffered from dementia. She was still functioning, mostly independently, but had noticed that keeping a schedule was more challenging, and her partner noticed repetitive questions. She had decided not to start the previously recommended donepezil, a medication that binds and irreversibly inhibits acetylcholinesterase, an enzyme that breaks down the chemical acetylcholine and ultimately increases acetylcholine concentrations at cholinergic synapses [7]. Donepezil was FDA-approved for the treatment of AD in 1996 [7].

Her physical exam was unremarkable, with no focal neurologic deficits. Although she was a little anxious during the visit, she did not exhibit any depression or anxiety symptoms. On her cognitive assessment, she scored zero out of five points on recall and lost two additional points for an overall score of 22/30; the normal cutoff score is 26. Her Quick Dementia Rating System (QDRS) score was a four on a continuous scale with a range of 0-30, supporting mild cognitive impairment [8]. Her workup included a lumbar puncture to evaluate her cerebrospinal fluid (CSF) for biomarkers of AD. Her CSF results were consistent with AD, with elevated phosphorylated tau (p-tau) and a low Aß42 to total tau (t-tau) index (ATI). This patient and her partner were now following up to see if there were any new medications that she could qualify for, as they had heard about new dementia medications that could slow down her decline.

For many other patients in this scenario, we might have considered monoclonal anti-amyloid medications. However, she reported unprovoked tonic-clonic seizures with onset during her teenage years. She had no additional medical conditions. Fortunately, her last seizure was over three decades ago, and she has remained treated with phenobarbital (64.8 mg daily) ever since her diagnosis, both suggesting relatively lower seizure risks. Still, clinical trials for monoclonal anti-amyloid medications excluded individuals with recent seizures (within the last 10 years [1], or 12 months [2]) or recurrent seizures [9]. While remote seizures would not have triggered trial exclusion, and published literature does not explicitly state how many patients with remote seizures were included, these patients were likely scarce amongst trial enrollees, and guidelines recommend against these medications in anyone with any history of seizures until additional data are available [3,4].

We ultimately decided to have her follow up with a neurologist and complete an electroencephalogram (EEG) for seizure risk stratification, with subsequent consideration of phenobarbital taper. This was due to the long duration of seizure freedom, suggesting a lower seizure recurrence risk, but also to phenobarbital’s potential multiorgan (and in this case, particularly cognitive) adverse effects. We discussed that even if cognition did not improve after possibly stopping her antiseizure medication (ASM), and even if she went on to fulfill the criteria for ‘resolved epilepsy’ (seizure-free for >10 years, with no ASMs for >five years) [10], appropriate use guidelines for lecanemab nonetheless still suggest against its use in patients with any history of seizures.

## Discussion

While there are virtually no exclusions listed on FDA labels against monoclonal anti-amyloid medications, appropriate use guidelines have still suggested caution in numerous groups [3,4]. One example pertains to patients with a seizure history. The concern is that approximately 15-35% of patients receiving anti-amyloid medications in trials experienced amyloid-related imaging abnormalities (ARIAs), which cause either edema or hemorrhage, rarely with serious adverse clinical outcomes. In aducanumab trials, 6/2,177 (0.3%) of patients in aducanumab arms had a seizure thought to be related to an ARIA, though only one of which was a generalized tonic-clonic seizure, and incidence was balanced between treatment versus placebo arms [11]. Still, while patients were includable in trials if their last seizure was >10 years [1] or >12 months ago [2], caution nonetheless exists regarding using anti-amyloid treatments in this group given the theoretical concern that they may be at higher risk of having a breakthrough seizure for the several-month acute period should an ARIA develop. However, given the likely extremely small numbers of patients with a history of seizures or epilepsy included in trials, an information gap remains regarding the risk of breakthrough seizures in this group.

AD and seizures represent an important bidirectional relationship [6,12]. The incidence of epilepsy is higher at older age than at any other time in life [13], its cumulative incidence doubles between the ages of 50 (1.7%) and 80 (3.4%) [14], and 20% of patients with AD have a seizure at some point [15]. Seizures can occur in the early and late stages of AD, leading to a decline in function and behavioral changes [16], and many older adults remain on first-generation ASMs (e.g., phenytoin, phenobarbital, and carbamazepine) [17] that have cognitive implications. This is particularly important given a past randomized trial of deprescribing mostly first-generation ASMs that suggested some cognitive improvement in the deprescribing arm [18]. National guidelines suggest consideration of ASM discontinuation in patients who become seizure-free after having a thorough risk-benefit discussion [19,20]. Though in practice, discussions surrounding ASM deprescribing are rare, particularly in older adults, despite many who might be low-risk even if deprescribed ASMs [19].

This case is meant to highlight the importance of discussions around seizure history and consideration of ASM discontinuation when appropriate. Most patients living with AD will not qualify for the new AD medications, given the limited application to individuals in the early stages of the disease and several other appropriate use criteria [3,4]. Recommendations suggest against using these medications in the setting of a large array of neurological comorbidities, particularly those with a history of transient ischaemic attack (TIA) or stroke within 12 months, significant microvascular disease, or intracranial hemorrhage or microhemorrhage [3]. Seizures represent just one of many neurological and other general medical reasons that clinicians may favor against using anti-amyloid medications. The key pertains to the risk-benefit assessment. This patient’s seizure risk was likely low given prolonged seizure freedom, a lack of a structural seizure etiology, and continued ASM treatment. Her seizure recurrence risk would be even lower if she had an EEG without epileptiform discharges, or if additional history confirmed her epilepsy diagnosis to be a self-limited childhood syndrome, or not epilepsy at all but rather febrile or other-provoked seizures or a seizure mimic. Additionally, ARIAs have been suggested to likely represent a short-term monophasic illness with radiographic resolution after the acute phase. The question remains: are there patients with prior seizures or other co-existing neurological disorders who might be considered for anti-amyloid treatment? Is it reasonable to consider these treatments for some subset of patients with a prior seizure who demonstrate enough favorable risk factors regarding seizure recurrence, or if their prior seizures were mild enough (e.g., nonconvulsive), if they are sufficiently treated with an ASM, or if the impact of AD is severe enough? If so, this may suggest a missed opportunity to treat an important subset of patients at particular risk for cognitive impairment.

Still, given the small magnitude of expected clinical efficacy, the steep costs of these new therapeutics, and the available alternative course of treatment, attempting to deprescribe her phenobarbital to assess for cognitive improvement seemed like a reasonable first step. Patients with concurrent neuropsychiatric disorders are likely to have multifactorial causes for cognitive impairment, including but also beyond AD (e.g., mood or sleep dysfunction, cerebrovascular disease, seizures, polypharmacy, etc.), that would not be addressed by anti-amyloid treatments.

## Conclusions

This patient story reminds readers of the complexities associated with the new anti-amyloid therapies for patients with AD, specifically as it relates to individuals with a prior history of seizures. This is an important group to consider as the therapeutic landscape of AD changes, given the intersection of AD and seizures in older adults. Even in exceptional candidates, clinicians may be wary of considering this low-benefit, potentially high-risk, high-cost intervention. Considering what has been published from clinical trials of these new disease-modifying AD medications and the groups excluded from these trials, clinicians should exercise caution while considering risk-benefit. A small excess risk may justifiably further dissuade a clinician from pursuing anti-amyloid treatments. This case highlights the importance of considering other therapeutic options and the potential benefits that may come from deprescribing medications if appropriate. Nevertheless, the need remains for high-quality data clarifying risk-benefit calculations amongst patients with existing neurological disorders, as these individuals may have the most to gain or lose from these new anti-amyloid medications.
